# Delivering a geriatric OSCE station in times of Covid-19 using makeup artistry

**DOI:** 10.3205/zma001382

**Published:** 2020-12-03

**Authors:** Daniel Bauer, Miria Germano, Johanna Stierlin, Beate Brem, Yvette Stöckli, Kai P. Schnabel

**Affiliations:** 1 University of Bern, Institute for Medical Education, Bern, Switzerland; 2 Siloah Akutklinik, Pflege und Rehabilitation, Gümligen, Switzerland

**Keywords:** geriatrics, OSCE, Covid-19, medical moulage, standardized patient

## Abstract

In the wake of the Covid-19 pandemic, people over 65 or suffering from certain conditions were deemed at high risk and asked to isolate themselves. This led to the simulated patient (SP) program at the University of Bern being depleted of middle-aged and elderly SP. Meanwhile, an OSCE had to be delivered using adapted cases that minimized physical contact between candidates and SP. Short of suitable cases at such short notice, the case of an elderly patient with postural instability had to be added to the exam blueprint. With elderly SP off the roster, it was decided to use makeup effects to achieve visual authenticity.

A combination of wigs (grey hair, hairdo), 3D Probondo transfers (forehead wrinkles), old age stipple (crow’s feet), and colouring (age spots) were used to achieve the old-age effects, while SPs wore scarves to cover their neckline. The lower face was covered with protective face masks in accordance with the exam’s Covid-19 hygiene protocol.

Case-related feedback from candidates and examiners was analysed for any direct or indirect remark attributable to the ageing effects. As no comment touched upon the subject of the appearance of age, this was interpreted as success, as any distracting effect from the SPs’ appearance in this regard would surely have prompted remarks or even complaints. The SPs’ feedback revealed how applying the ageing effects helped them adopt the octogenarian’s role.

This report explains how SP in their fifties were made fit for an octogenarian’s case in an OSCE using makeup effects. The effort required for the ageing simulation was considerable, but it is hoped that in future, with more planning time, the amount of effort required can be reduced. The feedback obtained from the candidates suggest the appearance of SPs was not experienced as a distraction, which was the primary objective of this exercise. Adapting our approach to their own contexts allows educators to include cases with elderly patients in their OSCE that cannot be re-written for younger SP, so long as Covid-19 prevents elderly SP from participating.

## Zusammenfassung

In der Covid-19-Pandemie wurden ein Lebensalter über 65 oder das Vorliegen bestimmter Gesundheitszustände als Risikofaktoren definiert und Betroffene entsprechend zur Selbstisolation aufgefordert. Dies führte auch dazu, dass das Simulationspersonenprogramm der Universität Bern auf den Einsatz vieler SP v.a. mittleren und vorgerückten Alters verzichten musste. Für eine unaufschiebbare OSCE-Prüfung wurden die Stationen derart angepasst, dass physische Nähe zwischen Kandidat*innen und SP minimiert war. Da sich in der kurzen zur Verfügung stehenden Zeit nur wenige Stationen derart anpassen liessen, musste der Fall einer älteren Patientin mit Haltungsinstabilität in den Prüfungsplan aufgenommen werden. Da ältere SP jedoch nicht aufgeboten werden konnten, wurde beschlossen, die visuelle Authentizität an dieser Station mittels Make-up-Effekten zu erzielen.

Die Alterungseffekte wurden mithilfe von Perücken (graues Haar, Frisur), 3D-Probondo-Transfers (Stirnfalten), Old Age Stipple (Krähenfüsse) und Schminke (Altersflecken) bewirkt. Schals bedeckten die Dekolletés und gemäss Covid-19-Schutzreglement wurden chirurgische Masken getragen.

Das stationsbezogene Feedback der Kandidat*innen und Prüfer*innen wurde auf direkte oder indirekte Bemerkungen zu den Alterungseffekten überprüft. Dass keine Äusserung bezüglich der altersgerechten Erscheinung der SP identifiziert werden konnte, wurde als Erfolg interpretiert, da jede ablenkende Wirkung des dargestellten Alters sicherlich zu Bemerkungen oder sogar Beschwerden geführt hätte. Das Feedback der SP zeigte ausserdem, wie die Alterungseffekte halfen, die Rolle der Achtzigjährigen anzunehmen.

Dieser Bericht erläutert, wie SP in ihren Fünfzigern mithilfe von Make-up-Effekten für die Rolle einer Achtzigjährigen in einem OSCE vorbereitet wurden. Der Aufwand für die Alterungssimulation war beträchtlich, aber mit mehr Planungszeit könnte dieser bei zukünftigen Anwendungen reduziert werden. Die Rückmeldungen der Kandidat*innen deuten darauf hin, dass die Erscheinung dieser SP nicht als Ablenkung empfunden wurde, was Hauptziel der Intervention war. Der beschriebene Ansatz lässt sich adaptieren, sodass auch andernorts Stationen mit älteren Patient*innen in OSCE aufgenommen werden können, wo eine Verjüngung der Rolle nicht infrage kommt, zumindest solange Covid-19 die Teilnahme älterer SP verhindert.

## Background

In Switzerland, when faced with the Covid-19 pandemic, a State of Extraordinary Circumstances in line with the Swiss Epidemics Act on 16 March 2020 was declared and measures to tackle the situation were issued. Next to rules for the general population, groups deemed to be at especially high risk were identified and encouraged to isolate themselves as much as possible to minimize their risk of infection. These groups included people over the age of 65 and people with specific conditions [1]. The measures gravely impacted medical schools, who had to reorganize in the middle of the term but specifically put simulated patient (SP) programs on the spot. The University of Bern’s SP program suddenly lost access to half of their 130 SP. Delivery of OSCEs had to be re-organized within the shortest of timeframes.

For the 3rd year OSCE at the University of Bern, a hygiene concept incorporating general rules (e.g. physical distancing, use of hand/ surface disinfectants, disposable gloves, and protective face masks) was developed. A redesign of the exam blueprint fell back on cases requiring no close physical contact between candidates and SP. This predicament led to the necessary inclusion of a geriatrics case, centred around a patient in her mid-eighties with a loss of postural stability, and that was to run in three parallel streams.

As an OSCE is a simulation-based format, three dimensions of authenticity were analysed, i.e. the narrative’s plausibility (case scenario and script), authenticity of environmental stimuli (physical surroundings being in line with the scenario), as well as the patient representation (i.e. the SP and their performance) [2]. As case script and checklist were adapted and with the exam to take place in a former hospital, what remained was seeing that the available SP would be able to enact the given role. It was argued that SP in their fifties, being the closest fit, would constitute a distracting visual stimulus with the possibility of diminishing the candidates’ engagement. This discrepancy in ages was to be tackled with special effects.

**Figure 1:**
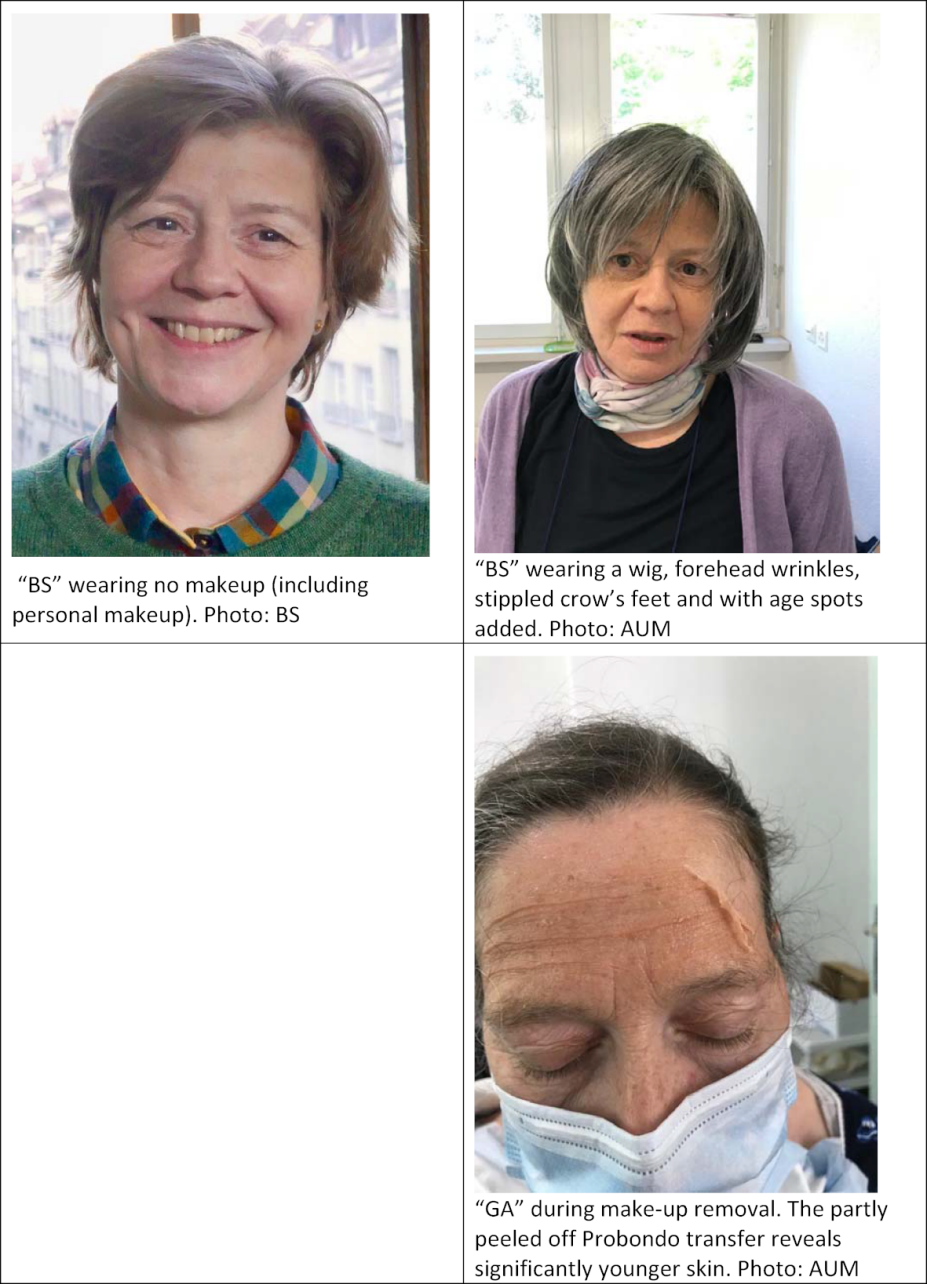
Photo documentation before and after the application of aging effects, and when removing make-up.

This article describes the measures taken to transform 4 SPs in their mid-fifties into geriatric patients in a standardized way with special ageing effects makeup (see figure 1 (Fig. 1)).

## Methods

Using current photos, the 4 SPs’ hair, face, neckline, and hands were analysed. The use of transfer techniques [3] would make the most of the little time in the OSCE’s morning and ensure a high degree of standardization. To save time, necklines were to be covered with scarves and effort on the SPs’ hands was reduced. Since the SP were to wear face masks during the exam, focus was put around the eyes as well as hair colour and style. Flat moulds of forehead wrinkles were modelled and created as 3D Probondo transfers (maekup, Faversham, UK), adding age spots in the colouring. A selection of age-appropriate wigs was requisitioned. 

For application, 1 makeup artist and 2 assistants were at the ready, and the 4 SP were to arrive early in a 1,5 h staggered schedule, not wearing any own makeup. Wigs were fitted and assigned. Crow’s feet, and further wrinkles were realized around the temples, cheekbones and below the eyes with latex-based “Old Age Stipple”. Forehead wrinkle transfers were adapted to the SP’s own skin tone, then applied and sealed.

When necessary, dark rings under the eyes were emphasized using grease-based colours, and further age spots were added around the cheekbones using alcohol-based colours. A thin line of red kajal applied to the lower eyelids suggested dry eyes, a common condition in elderly. If warranted, eyebrows were additionally greyed in, to better match the wig’s colour. Finally, SPs put on their wigs and scarves and joined the exam’s main program in time. Removal of makeup took around 30 minutes per SP after the exam.

The ageing effects administered were not evaluated explicitly to avoid cuing, but feedback from candidates (n=199) and examiners (n=3, from the case’s three parallel streams) was collected as usual in assessment contexts. Examiners and SP were contacted post-hoc to share their experiences. This feedback is shared narratively.

## Results

The case-related feedback from candidates and examiners was analysed for direct or indirect remarks attributable to the ageing effects. Neither the examiners covering the geriatric case, nor any of the 20 candidate remarks submitted commented on the ageing effects administered. Examiners post-hoc feedback was that they regarded the SPs’ visual appearance as fitting the case.

All four SP provided post-hoc feedback. Statements include how authentic makeup helped them adopt their role. One SP shared how she first had reservations about sharing the makeup room with other people during the ongoing pandemic but relaxing when she noticed everyone following the preventive measures. One SP complained of a minor pinching feeling from wearing the wig. One SP describes how she was happy appearing “just old” instead of “a caricature of old”. Another bemoaned how her hair was deemed grey enough as not to warrant a wig and how she still felt too young with all effects in place but pictured her own mother and how that mindset helped her adopt an octogenarian posture. Two speculate on the dominant role of the wigs and how without them candidates probably would have engaged less naturally. Makeup removal was commonly described as an alleviating experience, seeing the rejuvenation as pleasurable, while one SP reported minor skin irritation from make-up removal.

## Discussion

We outlined how SP in their fifties can be turned into octogenarians in an OSCE using makeup effects. The visual must be accompanied by age-appropriate postural, vocal, and cognitive behaviour. Effort put into the ageing simulation was considerable, but could be reduced with more planning time. The goal was not for the SP to transform into authentic octogenarians but to appear so as not to undermine the candidates’ simulation engagement by looking confusingly young.

Although the ageing effects were not specifically evaluated, candidates in exams tend to be wary and would rather report anything extraordinary than not. Combined with the examiners’ feedback, we hold the SPs’ visual age was not experienced as distraction. 

It remains unclear how the Covid-19 pandemic will play out. Assuming there will still be a place for face-to-face simulation-based exams, and that especially elderly SP might not be available for some time being, the approach described allows for educators to include cases with elderly into an OSCE that cannot be re-written for younger SP.

## Competing interests

The authors declare that they have no competing interests.
